# New Frontiers in Endodontics: Tackling the Mesiobuccal 2 (MB2) Canal Challenge in the Maxillary First Molar

**DOI:** 10.7759/cureus.59881

**Published:** 2024-05-08

**Authors:** Khyati Manik, Anuja Ikhar, Aditya Patel, Manoj Chandak, Jay Bhopatkar, Mrudula Shinde

**Affiliations:** 1 Conservative Dentistry and Endodontics, Sharad Pawar Dental College and Hospital, Datta Meghe Institute of Higher Education and Research, Wardha, IND; 2 Orthodontics and Dentofacial Orthopaedics, Sharad Pawar Dental College and Hospital, Datta Meghe Institute of Higher Education and Research, Wardha, IND

**Keywords:** biomechanical preparation, obturation, access opening, maxillary first molar, mb2 root canal

## Abstract

Molars have been observed to have a variety of morphological forms. The least understood and most treatable tooth in the posterior region is the upper first molar. The maxillary first molar has a complex anatomy with a wide variation in the number, size, and shape of the root canals. The case documentation highlights the endodontic treatment of the upper first molar with an anomalous position of the mesiobuccal 2 (MB2) canal. Endodontic therapy success is contingent upon the awareness of the dentist about the differences i.e. morphology and outline of the root and its canal.

## Introduction

A fundamental insight into the root and its canal morphology is essential for the effective treatment of the root canal [1]. All root canals must be recognized, and proper biomechanical preparation, irrigation, and obturation should be done to meet the major goals of endodontic treatment [2]. According to reports, the upper first molar typically has three to four canals and three roots [3]. Numerous approaches for analyzing the variation of the upper first molar's mesiobuccal root have been published in the literature. Clinical, radiological, and histopathological studies such as “clearing”, “staining”, and “sectioning” are examples of these approaches [4].

One of the best diagnostic image modalities that produce good quality and detailed reconstruction of the components of the maxillary and facial skeleton that are mineralized is cone beam computed tomography. There are many setups of cone beam computed tomography available that give radiographic pictures with a small field of view, low dose, and high spatial resolution for endodontic diagnosis, evaluation of post-treatment, and therapeutic guidance [5]. Although panoramic and periapical radiographies give satisfactory features in the following directions, i.e. mesial and distal, details in the buccal-lingual dimension are insufficiently observed. As a result, it is critical to get multiple X-rays with varying angulations [6].

Additionally, the use of microscopic endodontics enhances visibility during procedures, enabling more precise identification and navigation of complex canal morphology, including the mesiobuccal 2 (MB2) canal. Ultrasonic technology equipped with small, flexible tips allows for precise detection and exploration of hidden canals. Enhanced access preparation techniques and advanced instrumentation further aid in reaching and treating the MB2 canal effectively.

Endodontists are typically tasked with diligently seeking out an MB2 canal. Larger roots, often boasting greater bucco-lingual dimensions, tend to harbor an MB2 canal more frequently. Neelakantan et al. and Karunakar et al. stated that the prevalence of MB2 canal in upper first molars in the Indian population was 44.1% and 47.1%, respectively [7,8]. MB2 canal exhibits considerable variability in its location, curvature, and morphology. This variability makes it difficult to predict its exact position, especially in complex root canal systems. Additionally, inadequate access preparation or insufficient visibility due to calcifications, pulp stones, or other obstructions can hinder the identification of the MB2 canal orifices. Limited instrumentation and outdated techniques may also contribute to difficulties in locating the MB2 canal, particularly in cases where traditional methods fail to adequately explore the entire root canal system. There are higher chances of undiagnosed MB2 canals in cases of endodontic retreatment in comparison to the teeth that are treated for the first time. Therefore failing to detect, shape, and obturate the existing MB2 canals results in a poor prognosis [9].

## Case presentation

A 38-year-old female patient living in Wardha contacted the Department of Conservative Dentistry and Endodontics of Sharad Pawar Dental College and Hospital with a complaint of pain in the right upper back of the jaw for a month. When the chief complaint was specified, the pain was spontaneous or persisted for minutes after the stimulus (usually heat, less often cold) was removed. The past medical history as well as past dental history of the patient was non-significant. Extra oral, as well as intra-oral clinical examination, was done. On extra oral examination, there was no sign of swelling or asymmetry on the patient's face. On intra-oral oral examination, deep occlusal caries were seen with the upper right first molar with tenderness on percussion positive. A mobility test with 26 was performed which showed no detectable movement. There was no reported history of trauma, fever, swelling, or pus discharge. Radiolucency of the enamel, dentin, and pulp, as well as widening of the periodontal ligament space with the right upper tooth, were detected on X-ray examination. Pulp neurosensibility tests, such as electric pulp test and thermal test, were performed. After performing an electric pulp test, a delayed response of 16 was seen, and after performing a hot gutta percha test, 16 responded with pain which lingered on the removal of the stimulus.

**Figure 1:**
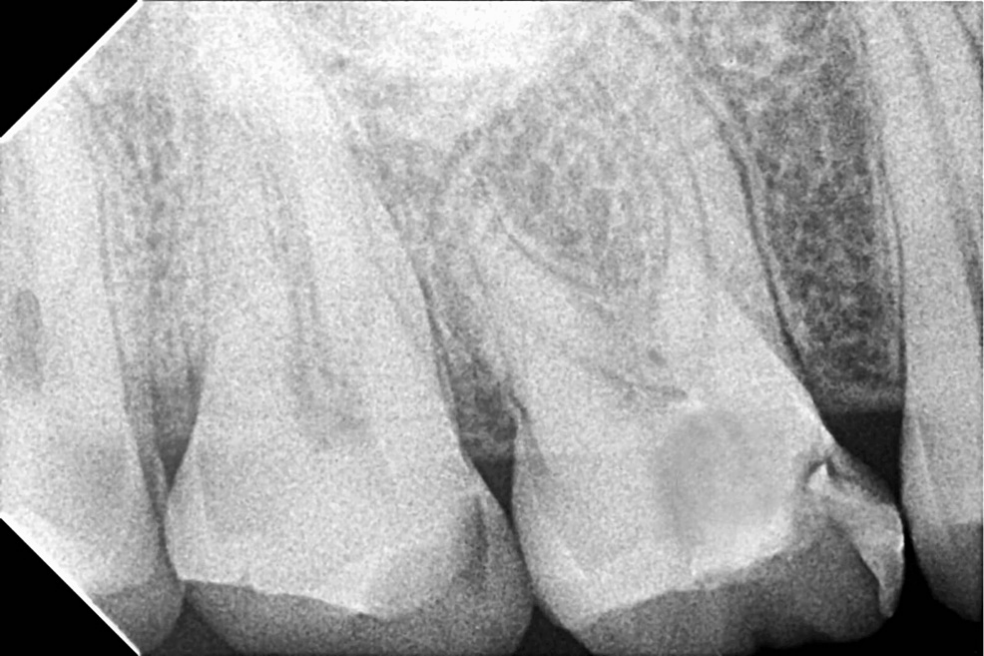
The figure shows a preoperative radiograph with the upper right first molar

As a result, a diagnosis of symptomatic irreversible pulpitis with apical periodontitis was made for the right upper first molar (Figure 1).

**Figure 2:**
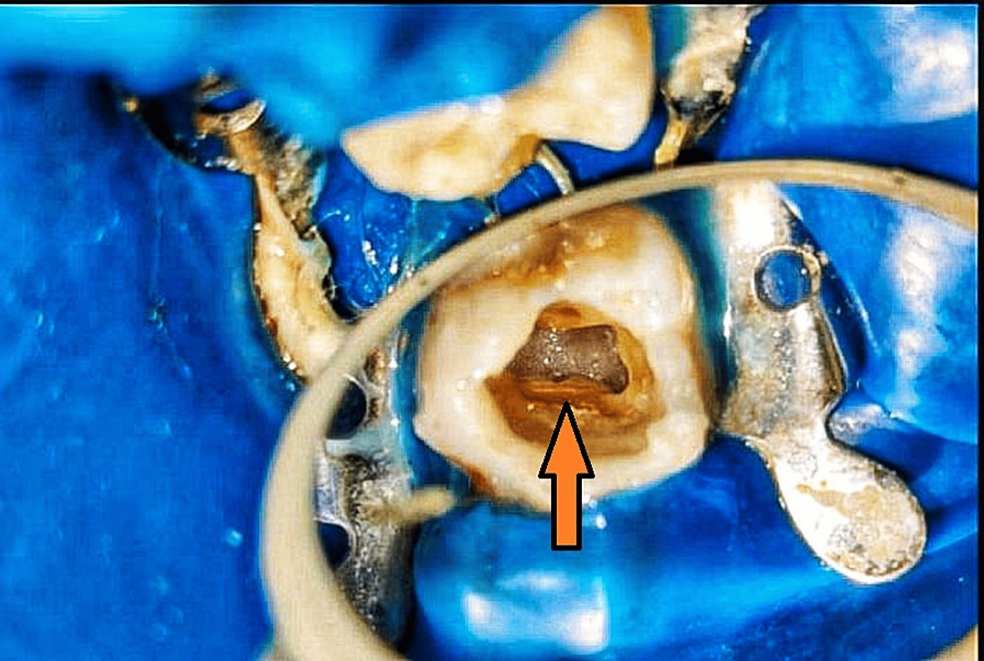
Access opening with the upper first right molar

The patient received 2% Xylocaine with 1:80,000 adrenaline (Xylocaine 2% injection; Zydus Cadila, Ahmedabad, India). A rubber dam isolation was done, and a 3.5x loupe was used for magnification and illumination. Round BR-45 (MANI, Utsunomiya, Japan) and safe end bur EX-24 (MANI, Utsunomiya, Japan) were used to prepare the access cavity. After removing pulp tissue from the chamber, three openings were discovered: palatal, mesiobuccal, and distobuccal openings in typical places. Using a combination of tactile exploration and enhanced visualization tools like 3.5x dental loupes or operating microscopes, the pulp chamber floor was probed especially from the mesiobuccal aspect where MB2 was found. Then the access cavity was modified by the removal of dentin in the mesiobuccal region, and widening of the cavity was done to enhance access to both the MB and MB2 canals. Throughout this process, proper irrigation with disinfectants is maintained to ensure visibility and cleanliness. The conventional triangular access was modified to a trapezoidal shape to improve access to the additional canal (Figure 2).

**Figure 3:**
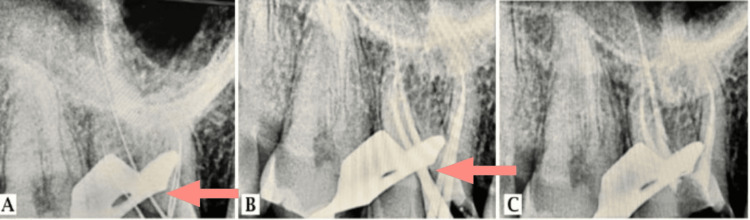
Radiographs of (A) working length, (B) master cone, and (C) obturation were taken using RVG RVG: radiovisiography

The working length was determined using Root ZX mini apex locator (J. Morita, Kyoto, Japan) and verified by taking an angled radiograph (palatal-21 mm, distobuccal-20.5 mm, mesiobuccal-20 mm, MB2-18.5 mm) (Figure 3A). Canals were cleaned and shaped using Rotary Ni-Ti (Woodpecker, Guilin, China) files up to 20 (4%) in MB2 canal, 20 (6%) in mesiobuccal and distobuccal, 25 (6%) in palatal. The canal was irrigated using 3% NaOCl and 0.9% saline alternatively. Calcium hydroxide temporary closed dressing (RC Cal; Prime Dental Products Pvt. Ltd., Thane, India) and temporary filling materials (Neotemp and Neoendo; Prime Dental Products Pvt. Ltd., Thane, India) were given and the patient was further recalled after seven days. On the second appointment, the patient was completely asymptomatic. The temporary dressing was removed, and all the canals were sonically activated with 17% EDTA using EndoActivator (Dentsply Sirona, Charlotte, USA) to facilitate calcium hydroxide removal. All the canals were then irrigated using 3% NaOCl and 0.9% saline alternatively. Gutta-percha master cones were selected for obturation (Figure 3B). The obturation process was carried out with master cones and epoxy resin-based sealer (Diaproseal; DiaDent Group International, Burnaby, Canada) using a cold lateral compaction technique, and X-rays were captured by radiovisiography (RVG) assistance (Figure 3C).

**Figure 4:**
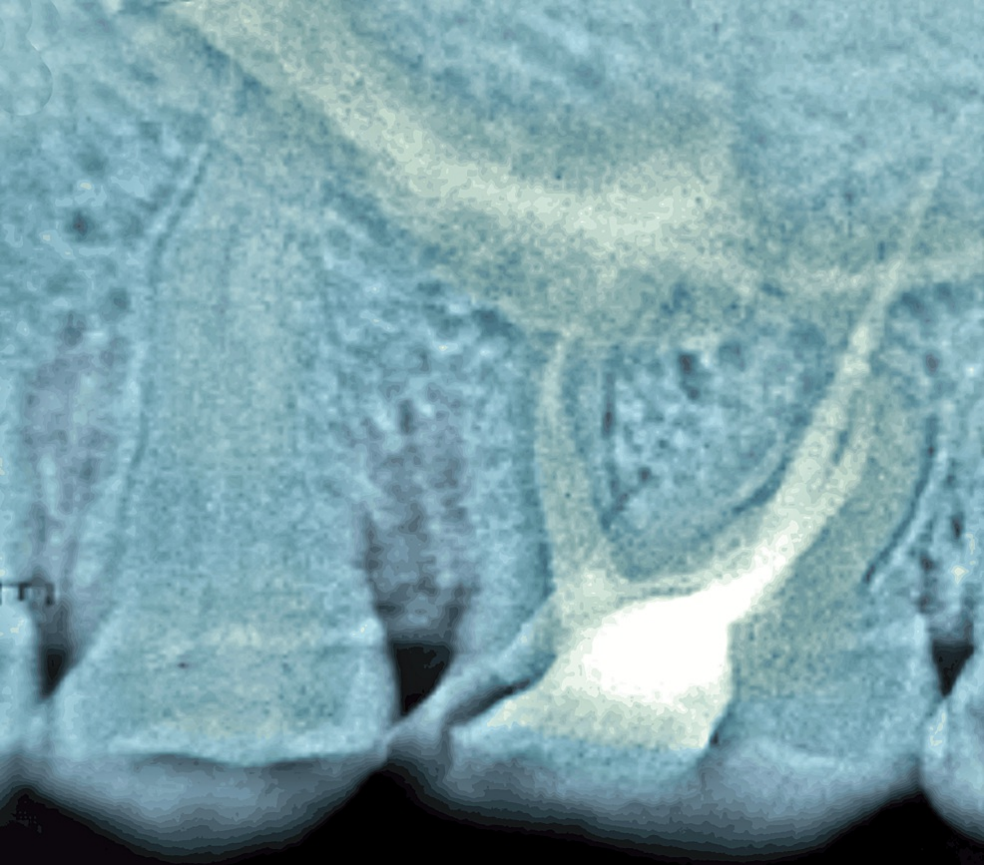
Post-obturation radiograph

The post-endo composite restoration (Spectrum; Dentsply Sirona, Charlotte, USA) was done (Figure 4).

## Discussion

Morphological variations in both the root canal and roots of teeth having multiple roots, continue to provide a diagnostic and therapeutic difficulty, and Weine et al. developed the most clinically useful morphological classification of the root canal system [10]. According to Tachibana and Matsumoto, the usage of computed tomography in the arena of dentistry allows for the inspection of the configuration of the root and their canals, and aspects of the tooth from all angles [11]. In line with this approach, the incidence of MB2 in the mesiobuccal root of the upper first molar tooth continues to be a cause of concern in the field of endodontics. The prognosis would be worse if the MB2 canal was not identified and treated [12].

According to the research of Peters et al., the distance between the first and second mesiobuccal canals ranges from 0.3 to 3.8 mm [13]. According to Kulild and Peters and Gilles and Reader, the mean distance from the first mesiobuccal canal to the MB2 canal is 2.31 mm and 1.82 mm, respectively [14,15]. The closer the openings are to each other, the more likely it is that the two root canals inside the root body will eventually join. Stropko discovered that by arranging an appropriate time for scientific purposes, using the most latest magnification and detection instrumentation aids, and having a complete understanding of methods for searching the MB2 canal, the incidence of locating the MB2 canal in the upper right first molar tooth can approach 93% [16]. In contrast to the triangular form, a rhomboidal access preparation should be made. A proper view of the region is made slightly mesial to a line that is drawn from the “mesiobuccal opening to the palatal opening”, allowing for the required preparation toward the mesial direction.

In the present case, 3.5x dental loupes are used for magnification and illumination purposes. The magnification provides a significantly enlarged and clearer view of the treatment area. This enhanced visualization allows clinicians to identify subtle anatomical features and distinguish between canal orifices more effectively. Magnification tools, such as dental loupes or operating microscopes, enable clinicians to work with greater precision. This is particularly crucial when modifying the access cavity to locate the MB2 canal, as it allows for the precise removal of dentin without damaging surrounding structures. It facilitates the recognition of anatomical variations such as isthmuses, fins, and accessory canals, which may be associated with the MB2 canal. Identifying these variations is crucial for thorough root canal treatment and preventing treatment failure due to missed canals. By providing a clearer view and enhancing precision, magnification reduces the risk of procedural errors such as perforation and ledging.

The orifice of MB2 is located on the groove that joins the palatal and mesiobuccal canals at a variable distance from later. The location of the MB2 orifice is approximately 2 mm lingual to the mesiobuccal orifice. Sometimes, this orifice is along a direct line between the mesiobuccal and palatal canals. Most often, it is mesial to the line connecting these two canals, appearing to be under the mesial marginal ridge. Troughing was done mesiopalatally and apically, along the mesiobuccal pulpal groove with distinct mesial orientation for locating MB2. The conventional triangular access was modified to a trapezoidal shape to improve access to the additional canal.

A discolored point lingual to the mesiobuccal canal may also be an indicator of the MB2 orifice. As soon as the minute point is discovered, meticulous planning of the chamber's mesial wall may disclose the MB2 canal's mesial course shortly before it courses apically [17]. Following the identification of the MB2 canal opening, the usage of long shank small round drills or different sizes of mesially inclined ultrasonic reamer tips (Woodpecker, Guilin, China) allows the upper calcified tissue to be opened by accessing the roof.

## Conclusions

The persistent challenge of missed MB2 canals in endodontics underscores the importance of implementing comprehensive strategies to enhance their detection and management. Employing a meticulous and systematic approach during root canal treatment, including thorough exploration of the pulp chamber and meticulous instrumentation, can increase the likelihood of locating and effectively treating MB2 canals. Furthermore, the development and utilization of innovative techniques and instruments tailored specifically for negotiating complex canal systems, such as ultrasonic activation and rotary instrumentation, offer additional means of addressing missed MB2 canals. These tools, coupled with enhanced magnification and illumination systems, afford clinicians unparalleled precision in accessing and treating subsidiary canals, mitigating the risk of missed MB2 canals and subsequent treatment failure. By doing so, clinicians can optimize the quality and longevity of endodontic treatment outcomes, ultimately benefiting the overall oral health and well-being of patients.
